# HDAC8-selective inhibitor PCI-34051 protects against aortic dissection by attenuating ferroptosis of vascular smooth muscle cells

**DOI:** 10.1093/lifemedi/lnag013

**Published:** 2026-04-17

**Authors:** Jiannan Ye, Juan Shi, Xin Yi, Jingjie Chen, Yi He, Bo Huo, Hanshen Luo, Shibin Chen, Xiang Wei, Ding-Sheng Jiang, Ze-Min Fang

**Affiliations:** Division of Cardiovascular Surgery, Tongji Hospital, Tongji Medical College, Huazhong University of Science and Technology, Wuhan 430030, China; Division of Cardiovascular Surgery, Tongji Hospital, Tongji Medical College, Huazhong University of Science and Technology, Wuhan 430030, China; Department of Cardiology, Renmin Hospital of Wuhan University, Wuhan 430030, China; Division of Cardiovascular Surgery, Tongji Hospital, Tongji Medical College, Huazhong University of Science and Technology, Wuhan 430030, China; Division of Cardiovascular Surgery, Tongji Hospital, Tongji Medical College, Huazhong University of Science and Technology, Wuhan 430030, China; Division of Cardiovascular Surgery, Tongji Hospital, Tongji Medical College, Huazhong University of Science and Technology, Wuhan 430030, China; Division of Cardiovascular Surgery, Tongji Hospital, Tongji Medical College, Huazhong University of Science and Technology, Wuhan 430030, China; Division of Cardiovascular Surgery, Tongji Hospital, Tongji Medical College, Huazhong University of Science and Technology, Wuhan 430030, China; Division of Cardiovascular Surgery, Tongji Hospital, Tongji Medical College, Huazhong University of Science and Technology, Wuhan 430030, China; Key Laboratory of Organ Transplantation, Ministry of Education, Wuhan 430030, China; NHC Key Laboratory of Organ Transplantation, Wuhan 430030, China; Key Laboratory of Organ Transplantation, Chinese Academy of Medical Sciences, Wuhan 430030, China; Division of Cardiovascular Surgery, Tongji Hospital, Tongji Medical College, Huazhong University of Science and Technology, Wuhan 430030, China; Key Laboratory of Organ Transplantation, Ministry of Education, Wuhan 430030, China; NHC Key Laboratory of Organ Transplantation, Wuhan 430030, China; Key Laboratory of Organ Transplantation, Chinese Academy of Medical Sciences, Wuhan 430030, China; Division of Cardiovascular Surgery, Tongji Hospital, Tongji Medical College, Huazhong University of Science and Technology, Wuhan 430030, China

## Abstract

Aortic dissection (AD) is a fatal emergency which lacks effective drug therapies. Previous studies demonstrated that histone deacetylase 8 (HDAC8) inhibition provides protective benefits in several cardiovascular diseases, including heart failure, fibrosis, and cardiac hypertrophy. However, the role of HDAC8 in AD remains unclear. In the present study, we investigated the function of PCI-34051, a highly selective inhibitor of HDAC8, in human aortic smooth muscle cell (HASMC) ferroptosis and **β**-aminopropionitrile (BAPN)-induced AD in mice. The results showed that PCI-34051 and HDAC8 knockdown significantly inhibited cystine deprivation (CD)- and imidazole ketone erastin (IKE)-induced HASMC ferroptosis, as evidenced by an increase in cell viability, reduction in cell injury/death, and lipid peroxidation levels in HASMCs. Transcriptome sequencing analysis revealed that the anti-ferroptosis effect of PCI-34051 was associated with the regulation of activator protein-1 (AP-1). Additionally, co-immunoprecipitation results showed that HDAC8 interacts with c-JUN, a component of AP-1. Overexpression of AP-1 (c-FOS and c-JUN) largely abolished the inhibitory effects of PCI-34051 on HASMC ferroptosis. More importantly, PCI-34051 reduced BAPN-induced AD incidence and aortic rupture mortality in mice by inhibiting HASMC ferroptosis and inflammatory response. Taken together, inhibition of HDAC8 by PCI-34051 may provide a preventive or therapeutic strategy for AD by attenuating HASMC ferroptosis.

## Introduction

Aortic dissection (AD) is a critical cardiovascular disease caused by a tear in the intima of the aortic wall, resulting in separation of the media and adventitia. To date, emergency surgery is still considered the gold standard of treatment. Nevertheless, the mortality rate for surgery intervention is still as high as 20%, even in large heart centres [1], underscoring the urgent need to develop effective pharmacological interventions for the prevention and therapy of AD. As is acknowledged, the loss of vascular smooth muscle cells (VSMCs) caused by ferroptosis and resultant aorta medial degeneration is the important pathological characteristic of AD [2]. Consequently, targeted inhibition of VSMC ferroptosis is a promising strategy for the treatment of AD.

Ferroptosis is a form of programmed cell death characterized by iron overload triggered intracellular lipid peroxidation [3]. In our previous studies, we have identified that VSMC ferroptosis was a novel pathological mechanism in AD. We demonstrated that VSMC ferroptosis was significantly activated during the formation of AD, and inhibitor of ferroptosis largely reduced β-aminopropionitrile (BAPN)-induced AD mortality and the incidence of aortic dilation in mice [4]. Furthermore, in our latest studies, we found post-translational modifications of histones were strongly correlated with VSMC ferroptosis and progression of AD. For example, we demonstrated that the histone methyltransferase inhibitors BRD4770 and SP2509 significantly inhibited VSMC ferroptosis and BAPN-induced AD in mice [4, 5]. Moreover, in the aorta of Stanford type A aortic dissection (TAAD) patients, we have also identified a reduction in the protein level of histone deacetylase and significant alterations in the acetylation levels of histones [6]. *In vitro* experiments have also shown that deficiency of histone acetyltransferase P300 accelerates ferroptosis in VSMCs through the HIF-1α/HMOX1 axis [7]. These findings suggest that the targeting of histone acetylation may be an effective therapeutic strategy for AD. However, the relationship between histone acetylation and ferroptosis, as well as AD, needs to be further elucidated.

A growing number of evidence suggests that the inhibition of histone deacetylases (HDACs) activity by HDAC inhibitors (HDACis) may offer therapeutic benefits in a variety of diseases [8]. HDAC8, the most recently recognized class I HDAC, is an attractive therapeutic target [9–11]. The majority of class I HDACis have been demonstrated to act as inhibitors of HDAC8. Nevertheless, it has also been observed that their inhibition of HDAC8 is frequently accompanied by inhibition effects on other HDACs [8]. PCI-34051, a potent and highly selective inhibitor of HDAC8, was derived from a low molecular weight hydroxamic acid scaffold, and comprises three structural modules: a zinc‐binding group (ZBG), a ‘linker’ moiety, and a ‘cap group’ [12, 13]. It showed a more than 200-fold greater selectivity on HDAC8 compared with other HDACisoforms [13]. PCI-34051 was reported to attenuate the inflammatory response in the cardiomyocytes to alleviate heart failure [11], and our previous study found that inflammatory response is an important driver of ferroptosis in VSMCs and AD [4]. It was therefore postulated that PCI-34051 could be associated with ferroptosis and AD. Further investigation of the effects of PCI-34051 on VSMC ferroptosis and AD is meaningful for the development of promising therapeutic agents for AD.

To investigate the relationship among PCI-34051, VSMC ferroptosis, and AD, we first established the VSMC ferroptosis models induced by cystine deprivation (CD)- and imidazole ketone erastin (IKE), and then determined the effect of PCI-34051 on cell viability and the levels of lipid peroxidation under the indicated conditions. To rule out potential off-target effects, we also examined the impact of HDAC8 knockdown on VSMC ferroptosis. Subsequently, RNA sequencing was used to elucidate the mechanism by which PCI-34051 regulated ferroptosis of VSMCs. Finally, a murine AD model induced by BAPN was applied to determine the effect of PCI-34051 on AD progression.

## Results

### PCI-34051 protects HASMCs from ferroptosis

**Figure 1.**
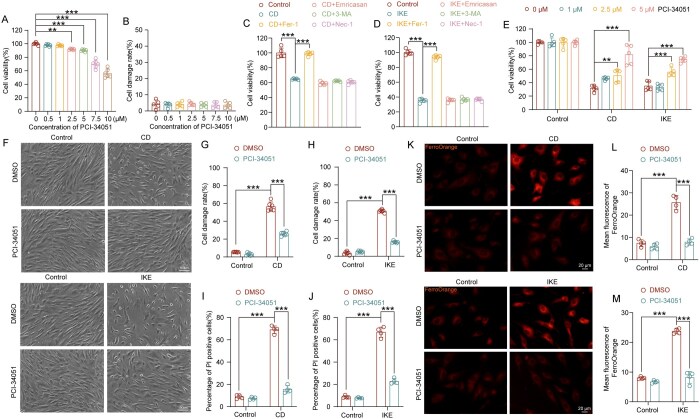
PCI-34051 inhibited CD- and IKE-induced ferroptosis in HASMCs. (A) Relative cell viability of HASMCs measured with a CCK8 kit after treatment with different concentrations of PCI-34051 (0, 0.5, 1, 2.5, 5, 7.5, and 10 μM) for 24 h (*n* = 6 per group). (B) Relative cell damage rate of HASMCs measured with an LDH kit after the same treatment above (*n* = 5 per group). (C, D) Relative cell viability of HASMCs measured with a CCK8 kit after cystine deprivation (CD) or imidazole ketone-erastin (IKE) induction and treatment with ferrostatin-1 (Fer-1, 2.5 μM), emricasan (5 μM), 3-methyladenine (3-MA, 5 mM), or necrostatin-1 (Nec-1, 10 μM) (*n* = 5 per group). (E) Relative cell viability of HASMCs measured with a CCK8 kit after CD or IKE treatment and different concentrations of PCI-34051 (0, 1, 2.5, and 5 μM) (*n*  =  5 per group). (F) Representative images showing cell death after treatment with DMSO and PCI-34051 (5 μM) under the ferroptosis models induced by CD and IKE. (G, H) Relative cell damage rate of HASMCs evaluated by LDH after treatment as described above (*n* = 6 per group). (I, J) Percentage of PI-positive examined by flow cytometry with propidium iodide (PI) staining after treatment as described above (*n* = 4 per group). (K) Representative images of FerroOrange fluorescence staining in HASMCs under the conditions as described above. (L, M) Quantitative analysis of FerroOrange fluorescence staining (*n* = 4 per group). Values are means ± SD; ***P < 0.001, **P < 0.01.

To investigate the effect of PCI-34051 on HASMC ferroptosis and determine the optimal drug concentration, we initially treated HASMCs with different concentrations of PCI-34051 (0, 0.5, 1, 2.5, 5, 7.5, and 10 µM) and examined the viability and damage rate of the cells with CCK8 and LDH assay. The results of CCK8 showed that PCI-34051 concentrations over 7.5 μM led to a notable decline in cell viability (Fig. 1A). While, the results of LDH assay indicated that PCI-34051 did not cause significant damage to the cells even at the concentration of 10 µM (Fig. 1B). To determine whether CD- and IKE-induction leads to specifically ferroptosis in HASMCs rather than other forms of programmed cell death, we treated HASMCs with 2.5 μM ferrostatin-1 (Fer-1, a ferroptosis inhibitor), 5 μmol/L emricasan (an apoptosis inhibitor), 5 mM 3-methyladenine (3-MA, an autophagic cell death inhibitor), or 10 μM necrostatin-1 (Nec-1, a necroptosis inhibitor). Only Fer-1 treatment significantly attenuated the CD- or IKE-induced reduction in HASMCs viability, whereas emricasan, 3-MA, and Nec-1 showed no notable protective effect (Fig. 1C and 1D). These results confirm that CD and IKE indeed induce ferroptosis in HASMCs. We next simultaneously stimulated CD- or IKE-induced HASMCs with different concentrations (0, 1, 2.5, and 5 µM) of PCI-34051. As shown in the results, PCI-34051 at a concentration of 2.5 µM and above significantly reversed the reduction in cell viability caused by CD and IKE in HASMCs (Fig. 1E). Accordingly, 5 µM PCI-34051 was selected for the following experiments. HASMCs were then treated with CD or IKE in combination with 5 µM PCI-34051 to investigate the effects of PCI-34051 on ferroptosis. As presented in the microscopic images, PCI-34051 markedly reduced CD or IKE-induced cell death (Fig. 1F). Furthermore, LDH assay and flow cytometric analysis of PI staining were used to evaluate cell injury and death. Consistent with the above results, PCI-34051 largely reduced cell injury and death of HASMCs under the treatment of ferroptosis inducers (Fig. 1G–J). Moreover, the results of FerroOrange staining showed that PCI-34051 treatment significantly inhibited the CD- or IKE-induced intracellular ferrous iron accumulation in HASMCs (Fig. 1K–M). These results suggested that PCI-34051 inhibited VSMC ferroptosis induced by both CD and IKE treatment.

### PCI-34051 effectively attenuates lipid peroxidation

**Figure 2.**
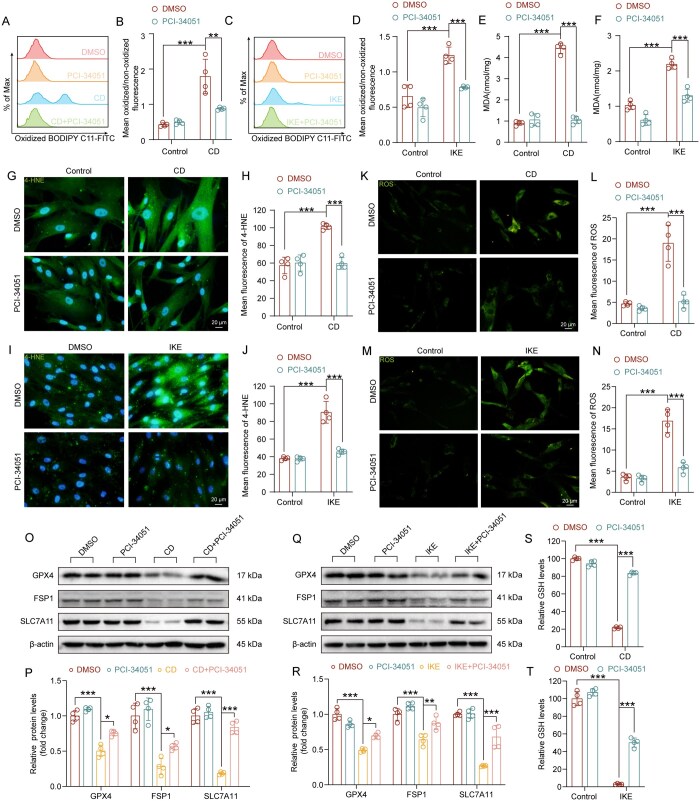
PCI-34051 reduced the accumulation of lipid peroxidation during HASMC ferroptosis. (A–D) The ratio of oxidized BODIPY-C11/non-oxidized BODIPY-C11 fluorescence examined by using a BODIPY-C11 kit after treatment with DMSO and PCI-34051 under the ferroptosis models induced by CD (A, B) and IKE (C, D) (*n* = 4 per group). (E, F) The level of MDA in HASMCs treated as described in (A–D) was measured with an MDA assay kit (*n* = 4 per group). (G, I) Representative images of 4-HNE immunofluorescence staining in HASMCs under the conditions described above. (H, J) Quantitative analysis of 4-HNE immunofluorescence staining (*n* = 4 per group). (K, M) Representative images of intracellular reactive oxygen species (ROS) staining with the DCFH-DA fluorescent probe in HASMCs under the conditions described above. (L, N) Quantitative analysis of ROS fluorescence staining (*n* = 4 per group). (O–R) Western-blot analysis and quantification showing the protein levels of GPX4, FSP1 and SLC7A11 in HASMCs after treatment with DMSO and PCI-34051 under the ferroptosis induction by CD and IKE. β-Actin served as a loading control (*n* = 4 per group). (S, T) Intracellular reduced glutathione (GSH) levels in HASMCs treated as described above were measured with a GSH Assay Kit (*n* = 4 per group). Values are means ± SD; ***P < 0.001, **P < 0.01, *P < 0.05.

Given that lipid peroxidation is a typical biological feature of ferroptosis [14], we proceeded to investigate the effects of PCI-34051 on lipid peroxidation during HASMC ferroptosis. BODIPY-C11, an oxidation-sensitive fluorescent lipid peroxidation probe, was used to assess the level of lipid peroxidation in HASMCs. As illustrated in Fig. 2A–D, the level of oxidized lipids in HASMCs induced by CD and IKE exhibited a notable increase, which was decreased significantly after treatment with PCI-34051, as evidenced by the decreased ratio of oxidized to non-oxidized BODIPY-C11 (Fig. 2A–D). Moreover, the level of malondialdehyde (MDA), a vital production and marker of lipid peroxidation, was markedly elevated in HASMCs treated with ferroptosis inducers, while PCI-34051 treatment largely reduced CD and IKE triggered MDA accumulation (Fig. 2E and 2F). Similarly, another important product of lipid peroxidation, the accumulation of 4-HNE, was markedly attenuated by PCI-34051 in HASMCs with CD and IKE treatment, as indicated by immunofluorescence staining (Fig. 2G–J). Subsequently, intracellular reactive oxygen species (ROS) were quantified using DCFH-DA fluorescent probe. The results showed that treatment with PCI-34051 significantly reduced ROS levels in HASMCs under ferroptosis-inducing conditions (Fig. 2K–N). Furthermore, we assessed the protein levels of key ferroptosis regulators involved in the redox signalling pathway using Western-blot analysis. The results revealed that induction of CD and IKE significantly decreased the expression of key ferroptosis regulators, including GPX4, FSP1, and SLC7A11. However, treatment with PCI-34051 notably restored the protein levels of these regulators (Fig. 2O–R). Additionally, as reduced glutathione (GSH) is a critical regulator in the redox pathway, we quantified its intracellular levels. It was observed that CD or IKE induction significantly depleted GSH in HASMCs, while treatment with PCI-34051 effectively preserved intracellular GSH content (Fig. 2S and 2T). These results revealed that PCI-34051 reduced lipid peroxidation accumulation during the ferroptosis of VSMCs.

### HDAC8 knockdown inhibits ferroptosis of HASMCs

**Figure 3.**
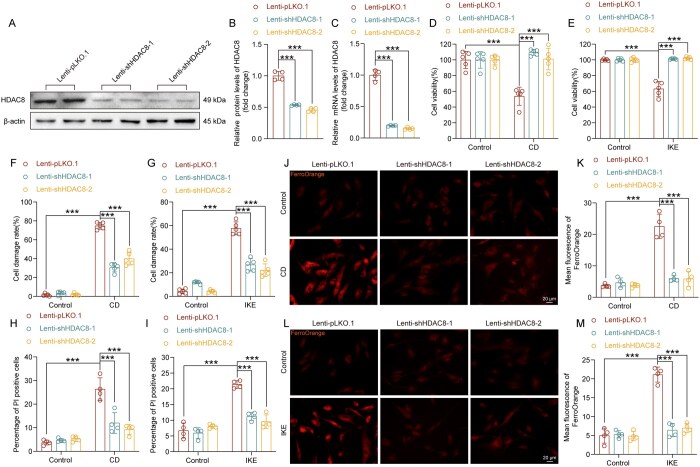
Knockdown of HDAC8 inhibited CD- and IKE-induced ferroptosis in HASMCs. (A–C) The protein and mRNA levels of HDAC8 were detected by Western blot and RT-PCR in HASMCs infected with lenti-pLKO.1 and lenti-shHDAC8 (*n* = 4 per group). (D, E) Relative viability of the indicated lentivirus-infected HASMCs evaluated by CCK8 after treatment with CD (D) and IKE (E) (*n* = 5 per group). (F, G) Relative cell damage of HASMCs infected with the indicated lentivirus was evaluated by LDH after the treatment described above (*n* = 5 per group). (H, I) Percentage of PI-positive indicated lentivirus-infected HASMCs examined by flow cytometry with propidium iodide (PI) staining after treatment as described above (*n* = 4 per group). (J, L) Representative images of FerroOrange fluorescence staining in HASMCs under the conditions described above. (K, M) Quantitative analysis of FerroOrange fluorescence staining (*n* = 4 per group). Values are means ± SD; ***P < 0.001.

**Figure 4.**
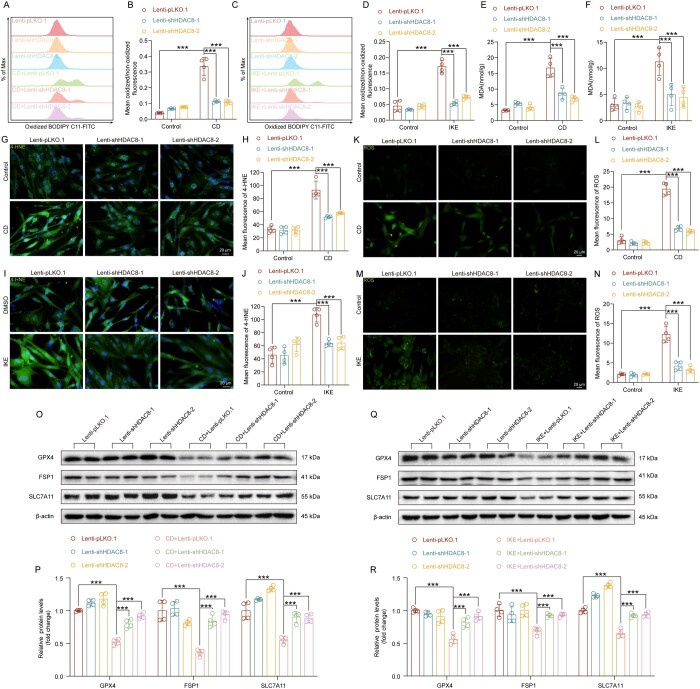
HDAC8 deficiency alleviated the accumulation of lipid peroxidation in HASMCs induced by CD and IKE. (A–D) The level of lipid peroxidation was examined by using BODIPY-C11 kit after treatment with CD (A, B) and IKE (C, D) after HASMCs infected with lenti-pLKO.1, lenti-shHDAC8-1, and lenti-shHDAC8-2 (*n* = 4 per group). (E, F) The level of MDA was evaluated by an MDA assay kit in indicated lentivirus-infected HASMCs after CD (E) and IKE (F) treatment for the indicated time (*n* = 4 per group). (G, I) Representative images of immunofluorescence staining of 4-HNE in the indicated lentivirus-infected HASMCs treated with CD (G) and IKE (I) treatment for the indicated time. (H, J) Quantitative analysis of 4-HNE (*n* = 4 per group). (K, M) Representative images of ROS staining with the DCFH-DA fluorescent probe in the indicated lentivirus-infected HASMCs after CD (K) and IKE (M) treatment. (L, N) Quantitative analysis of ROS fluorescence staining (*n* = 4 per group). (O–R) Western-blot analysis and quantification performed to assess the protein levels of GPX4, FSP1, and SLC7A11 in HDAC8-knockdown HASMCs after treatment with CD (O, P) and IKE (Q, R). β-Actin served as a loading control (*n* = 4 per group). Values are means ± SD; ***P < 0.001.

Although PCI-34051 is a highly selective inhibitor of HDAC8, the possibility of off-target effects cannot be completely ruled out. Therefore, we further evaluated the effects of HDAC8 knockdown on HASMCs ferroptosis. We first generated two distinct short-hairpin RNA (shRNA) plasmids targeting HDAC8 for gene knockdown (shHDAC8-1 and shHDAC8-2) (Fig. 3A–C). HASMCs infected with the indicated lentiviruses were then treated with CD or IKE to induce ferroptosis. The CCK-8 assay revealed that compared to the control, CD and IKE treatment reduced cell viability by approximately 50%, a reduction that was significantly alleviated by HDAC8 knockdown (Fig. 3D–E). Consistent with these results, the LDH assay and PI staining showed that HDAC8 deficiency notably protected against cell injury and death induced by CD and IKE (Fig. 3F–I). HDAC8 knockdown also effectively blocked the accumulation of ferrous iron in HASMCs induced by CD or IKE (Fig. 3J–M). Next, we assessed the effect of HDAC8 knockdown on lipid peroxidation in HASMCs. HDAC8 knockdown significantly attenuated CD- and IKE-induced lipid peroxidation in HASMCs, as demonstrated by BODIPY-C11 assay, MDA assay and 4-HNE immunofluorescence staining (Fig. 4A–J). Additionally, the increase in intracellular ROS levels caused by CD or IKE induction was effectively reversed by HDAC8 knockdown (Fig. 4K–N). Furthermore, Western-blot analysis showed that CD and IKE treatments substantially reduced the protein levels of anti-ferroptosis regulators GPX4, FSP1, and SLC7A11, while knockdown of HDAC8 restored their expression (Fig. 4O–R). These results revealed that knockdown of HDAC8 alleviated CD- and IKE-induced ferroptosis in HASMCs.

### The effect of PCI-34051 on HASMC ferroptosis is mediated by AP-1

**Figure 5.**
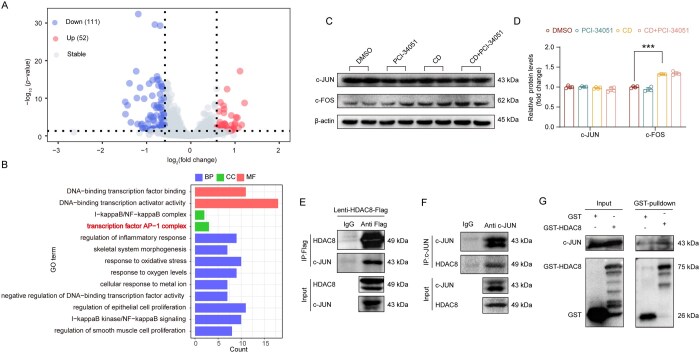
AP-1 (c-FOS and c-JUN) is a key regulator of PCI-34051 in the regulation of HASMC ferroptosis. (A) Volcano plot displaying the gene expression distribution and differentially expressed genes among the DMSO and PCI-34051 groups after treatment with CD. (B) Gene Ontology (GO) enrichment analysis showed that biological processes and cellular components were associated with these differentially expressed genes. (C, D) Western-blot analysis and quantification showing the protein levels of c-FOS and c-JUN in HASMCs after treatment with DMSO and PCI-34051 under the ferroptosis models induced by CD. β-Actin served as a loading control (*n* = 4 per group). (E, F) Co-immunoprecipitation results showed that HDAC8 interacted with c-JUN in HASMCs. (G) Results of GST pull-down demonstrated a direct interaction between HDAC8 and c-JUN in HASMCs. Values are means ± SD; ***P < 0.001. BP, biological process. CC, cellular component. MF, molecular function.

To gain further insight into the regulatory mechanism of PCI-34051 on HASMC ferroptosis, an analysis of RNA sequencing was performed on HASMCs treated with CD or PCI-34051 combined with CD. The differentially expressed genes were filtered out based on the threshold |log_2_(fold change)| ≥  0.585 and adjusted *P-*value ≤ 0.05 (Fig. 5A). The GO analysis was conducted to figure out the distinction of biological processes between the two groups. As shown in the results, terms related to transcription factors, particularly the AP-1 (activating protein-1) complex, were significantly enriched in addition to the pathways of oxidative stress, inflammation, which are closely related to ferroptosis (Fig. 5B). The transcription factor AP-1 is a dimeric complex composed of JUN, FOS, and ATF (activating transcription factor) families [15]. Therefore, we first determined whether the expression levels of c-FOS and c-JUN were regulated by PCI-34051 treatment. The results showed that PCI-34051 had no influence on the protein levels of c-FOS and c-JUN in HASMCs under CD treatment (Fig. 5C and 5D). Besides regulating expression, there are many regulators that can modulate the transcriptional activity of AP-1 by interacting with its subunits [16, 17]. Since PCI-34051 is an inhibitor of HDAC8, we are very interested in whether HDAC8 can interact with c-FOS or c-JUN to regulate the transcriptional activity of AP-1. The results of Co-IP showed that HDAC8 bound to c-JUN and *vice versa* (Fig. 5E and 5F), and the subsequent GST pull-down yielded consistent results (Fig. 5G).

**Figure 6.**
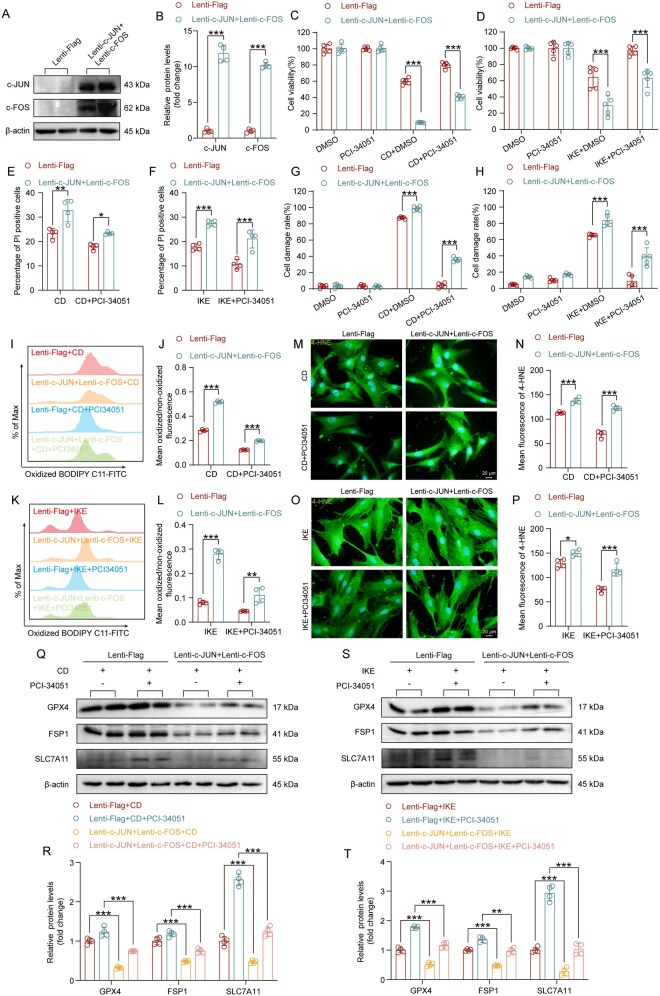
AP-1 (c-FOS and c-JUN) eliminated the effects of PCI-34051 on HASMC ferroptosis. All HASMCs were infected with lenti-Flag and lenti-c-FOS + lenti-c-JUN, and then these HASMCs were used for subsequent experiments. (A, B) The protein level of AP-1 (c-JUN and c-FOS) was detected by Western blot in HASMCs infected with lenti-Flag and lenti-c-JUN + lenti-c-FOS (*n* = 4 per group). (C, D) The CCK8 assay showing the relative viability of HASMCs treated with DMSO and PCI-34051 after CD (C) and IKE (D) stimulation for the indicated time (*n* = 5 per group). (E, F) Flow cytometry with propidium iodide (PI) staining displaying the percentage of PI-positive cells of HASMCs after treatment as described above (*n* = 4 per group). (G, H) The LDH assay indicating the relative cell damage rate of HASMCs treated with described above (*n* = 5 per group). (I–L) The ratio of oxidized BODIPY-C11/non-oxidized BODIPY-C11 fluorescence revealing the level of ROS of HASMCs treated with described above (*n* = 4 per group). (M–P). 4-HNE immunofluorescence staining and quantitative analysis exhibiting the content of 4-HNE in HASMCs after treatment as described above (*n* = 4 per group). (Q–T) Western-blot analysis and quantification performed to assess the protein levels of GPX4, FSP1, and SLC7A11 in AP-1 overexpressed HASMCs after DMSO and PCI-34051 treatment along with CD or IKE induction. β-Actin served as a loading control (*n* = 4 per group). Values are means ± SD; ***P < 0.001, **P < 0.01, *P < 0.05.

To further determine whether AP-1 (c-FOS and c-JUN) was associated with the ferroptosis inhibitory function of PCI-34051, the overexpression plasmids of c-FOS and c-JUN were constructed and used to infect HASMCs (Fig. 6A and 6B), then the cells were subjected to ferroptosis inducers combined with or without PCI-34051 treatment. As the results showed, compared to the control group, overexpression of AP-1 (c-FOS and c-JUN) significantly exacerbated CD- and IKE-induced decrease in cell viability, increase in cell damage and mortality (Fig. 6C–H). Similarly, compared with control, AP-1 (c-FOS and c-JUN) overexpressed HASMCs exhibited a higher level of lipid peroxidation in both CD and IKE treatment group, as evidenced by the elevated ratio of oxidized lipids and the levels of 4-HNE (Fig. 6I–P). Moreover, in contrast to the attenuating effect of PCI-34051 on the CD- or IKE-induced reduction in GPX4, SLC7A11, and FSP1 protein levels, AP-1 (c-FOS and c-JUN) overexpression led to further decrease of these proteins in HASMCs under ferroptosis induction (Fig. 6Q–T). In summary, AP-1 (c-FOS and c-JUN) greatly nullified the protective effect of PCI-34051 on ferroptosis and lipid peroxidation in HASMCs. Therefore, these results demonstrated that PCI-34051 prevents ferroptosis of VSMCs by suppressing the transcriptional activity of AP-1, which is achieved by HDAC8 directly interacting with c-JUN.

### PCI-34051 attenuated BAPN-induced AD through inhibiting ferroptosis in mice

**Figure 7.**
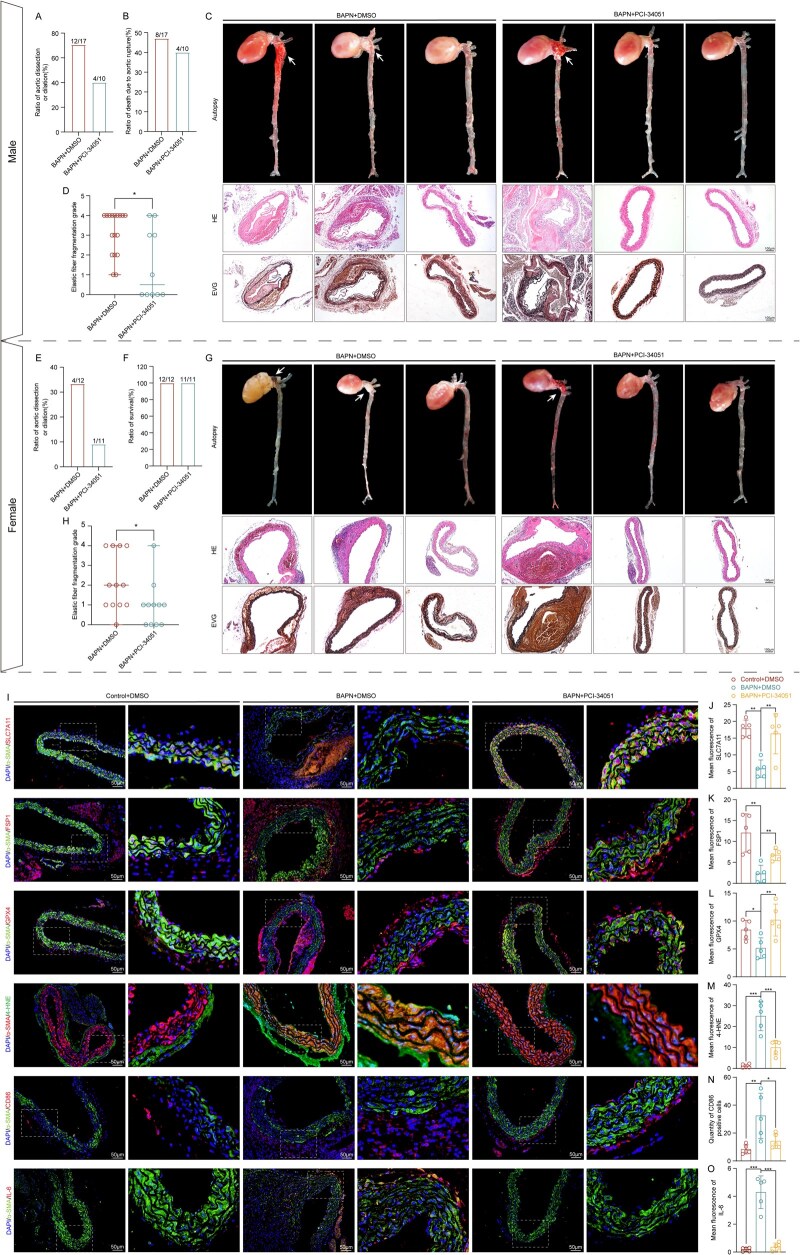
PCI-34051 alleviated the incidence of AD or aortic dilation in male and female mice. (A, B, E, and F) The incidence of AD or aortic dilation (A, E) and the mortality due to aortic rupture (B, F) of male (A, B) and female (E, F) mice in the BAPN + DMSO (male: *n* = 17 per group, female: *n* = 12 per group) and BAPN + PCI-34051 (male: *n* = 10 per group, female: *n* = 11 per group) groups after four weeks of treatment. (C, G) Representative images of excised aortae, haematoxylin and eosin (H&E), and EVG staining of male (C) and female (G) mice in BAPN + DMSO (male: *n* = 17 per group, female: *n* = 12 per group) and BAPN + PCI-34051 (male: *n *= 10 per group, female: *n* = 11 per group) groups after four weeks treatment. (D, H) The elastin rupture degree in aortic tissues of male and female mice in BAPN + DMSO (male: *n* = 17 per group, female: *n* = 12 per group) and BAPN+ PCI-34051 (*n* = 10 per group, female: *n* = 11 per group) groups. Values are presented as means with ranges; (I–O) Representative images and quantitative analysis showing immunofluorescence staining of SLC7A11, FSP1, GPX4, 4-HNE, IL-6, and the number of CD86-positive macrophages in aorta of DMSO, BAPN + DMSO, and BAPN + PCI-34051 groups (*n* = 5 per group). The representative images from the IgG control groups were presented in Fig. S2. ***P < 0.001, **P < 0.01, *P < 0.05.

According to our previous studies, VSMC ferroptosis plays an important role in the occurrence and development of AD [4, 5]. In order to verify the protective effect of PCI-34051 on AD *in vivo*, a mouse model of AD induced by BAPN was established in 3-week-old C57BL/6J mice, and PCI-34051 was injected intraperitoneally at a dose of 30 mg/kg every day, while the control group was given an equal volume of DMSO. The results showed that after four weeks of treatment, compared to DMSO, treatment with PCI-34051 resulted in a reduction in the incidence (70.59% vs. 40%) of BAPN-induced AD and mortality (47.06% vs. 40%) due to aortic rupture in male mice (Fig. 7A and 7B). As shown in the representative gross images, the primary sites of AD or aortic dilation at autopsy were observed in the ascending aorta and the arch (Fig. 7C). In addition, H&E and EVG staining of aortic tissue sections indicated that PCI-34051 effectively suppressed the degeneration of the aortic medial layer and the disruption of elastic fibres induced by BAPN (Fig. 7C and 7D). Current epidemiological evidence consistently demonstrates a significant sex disparity in AD incidence, with males exhibiting about 3-fold higher susceptibility compared to females (75% vs. 25% prevalence) [18]. To explore potential sex-specific therapeutic effects of PCI-34051, we conducted parallel experiments in female murine models. Quantitative analysis revealed that BAPN-treated female mice exhibited significantly lower dissection incidence (33.33% vs. 70.59%) and rupture-related mortality (0% vs. 47.06%) compared to their male counterparts (Fig. 7E and 7F). Importantly, PCI-34051 administration effectively suppressed BAPN-induced AD development in female mice, with histopathological validation through H&E and EVG staining showing preserved aortic wall integrity (Fig. 7G and 7H). In summary, these findings illustrate that PCI-34051 can reduce mortality and occurrence of AD, highlighting its potential therapeutic role in AD.

To substantiate the aforementioned hypothesis that PCI-34051 exerts a protective effect on AD by inhibiting ferroptosis of VSMCs, we employed immunofluorescence methodologies to evaluate the protein levels of key ferroptosis regulators and levels of lipid peroxidation products. Compared with the BAPN group, we found that PCI-34051 treatment significantly increased the protein levels of SLC7A11, FSP1, and GPX4 in the aortas of mice (Fig. 7I–L). Similarly, the lipid peroxidation product 4-HNE was markedly reduced in the aortas of mice with treatment of PCI-34051 compared with BAPN + DMSO group (Fig. 7I and 7M). The results suggested that PCI-34051 exerts an anti-ferroptotic effect in VSMC and prevents AD occurrence. In addition, AP-1 plays an important role in the regulation of the inflammatory response and the macrophage polarization, with evidence indicating that macrophage polarization is a crucial factor in ferroptosis and AD development [4, 19]. Thus, we further detected the expression levels of the inflammatory cytokine IL-6 and CD86, a key marker of M1 macrophages. The findings indicated a reduction in the levels of IL-6 and the number of macrophages with an M1 phenotype within the area of the aorta after PCI-34051 treatment, compared to the BAPN group (Fig. 7I, 7N and 7O). Taken together, PCI-34051 may serve as a potential therapeutic agent to attenuate the onset and development of AD by preventing VSMC ferroptosis and aortic inflammatory responses.

## Discussion

Aortic dissection is a devastating cardiovascular disease whose pathogenesis involves multiple pathological processes, including various factors contributing to medial degeneration, such as phenotypic switching and subsequent dysfunction of VSMCs, the loss of VSMCs, dysregulated extracellular matrix metabolism, and vascular immune-inflammatory responses. Ferroptosis of VSMCs constitutes a pivotal link in this cascade, which not only directly contributes to VSMC depletion but is also intricately linked to the pathological phenotypic switching of VSMC [20, 21]. Furthermore, lipid peroxides and reactive oxygen species generated during ferroptosis exacerbate extracellular matrix degradation [22], while ferroptosis also disrupts the homeostasis of immune cells, such as CD4^+^ T cells, thereby triggering and amplifying the local inflammatory response [23]. Increasing evidence revealed that VSMC ferroptosis represents a novel and important pathological mechanism of AD. In the present study, we revealed that both PCI-34051 and knockdown of HDAC8 exert a pronounced protective influence against ferroptosis in HASMCs. Furthermore, RNA sequencing analysis and subsequent experiments revealed that the inhibitory effect of PCI-34051 on HASMC ferroptosis was associated with the regulation of AP-1 (c-JUN and c-FOS). More importantly, our findings demonstrated that PCI-34051 reduced the incidence of AD and inhibited inflammatory response and VSMC ferroptosis in BAPN-induced AD mouse models.

Currently, HDACis have emerged as a novel therapeutic strategy in several diseases; six HDACis have been approved by the FDA for the treatment of cutaneous and peripheral T-cell lymphomas, multiple myeloma or neurological disorders [24]. However, research into the potential of HDACis in the treatment of cardiovascular diseases is still in the preclinical phase [25]. Previous studies suggested that HDACis, scriptaid (inhibitor of class IIa HDACs), TSA (inhibitor of class I, II, and IV HDACs), and RFGP966 (inhibitor of HDAC3) could inhibit the proliferation of VSMCs to prevent neointimal hyperplasia [26, 27]. Moreover, the class I HDACs inhibitor MS-275 and the class IIa HDACs inhibitor MC-1568 were found to significantly reduce the incidence and severity of abdominal aortic aneurysm (AAA) and limit aneurysmal expansion [28]. However, Zhang et al. [29] revealed that broad-spectrum pan-HDAC inhibitors, such as vorinostat and trichostatin A, increased the risk of thoracic AD and aneurysm, and exacerbated aortic elastin degradation and macrophage infiltration in mice. Overall, the role of HDACis in the context of cardiovascular disease remains a topic of contention, which could be attributed to the uncertainty of broad-spectrum inhibitor targets. It can be reasonably deduced that high specificity, in other words, single-targeted HDAC inhibitors, may yield superior outcomes. In the present study, we revealed the HDAC8-selective inhibitor PCI-34051 significantly attenuated the formation of AD and aortic dilation, which offers significant insights into the potential for AD treatment through targeting a single HDAC.

Ferroptosis is a distinctive mode of cell death driven by iron-dependent phospholipid peroxidation. Our investigation has led to the identification of an innovative role for PCI-34051 in the inhibition of VSMC ferroptosis. Given its involvement in a multitude of biological activities, ferroptosis is subject to regulation by a plethora of metabolic processes, including redox homeostasis, iron metabolism, inflammatory response, as well as several signalling pathways that are pertinent to disease [30]. One of our previous studies demonstrated that LPS-induced inflammatory response exacerbates VSMC ferroptosis [4]. Furthermore, several inflammatory pathways, including JAK-STAT, NF-κB, and MAPK, are implicated in the regulation of ferroptosis [31]. In accordance, PCI-34051 have also been revealed to ameliorate inflammation response in murine model of asthma [32]. The administration of PCI-34051 has been demonstrated to suppress STAT6 and PI3K/Akt signalling pathways to prevent M2 macrophage polarization in peritoneal fibrosis [33]. In the present study, a reduction in CD86-positive M1-polarized macrophages was observed in the aorta of mice treated with PCI-34051, which means PCI-34051 may inhibit ferroptosis of VSMCs through the modulation of inflammatory pathways. More importantly, our previous study demonstrated that the inhibition of ferroptosis in VSMCs markedly attenuated the progression of AD. These findings provide additional evidence for the regulatory role of PCI-34051 in VSMC ferroptosis and AD. Overall, our finding reveals that PCI-34051 offers a novel insight on the prevention and treatment of AD.

Our RNA-sequencing analysis data showed that the regulation of VSMC ferroptosis by PCI-34051 is associated with the activation of the AP-1 complex. AP-1, as transcription factors, are involved in a multitude of cellular life activities, including but not limited to proliferation, differentiation, and apoptosis, and have been reported to be associated with a variety of serious diseases [15, 34]. The association of AP-1 and ferroptosis has been elucidated in several studies. Ma et al. [35] demonstrated that the GPX4 promoter contained a putative AP-1 binding site, and c-JUN could inhibit *GPX4* transcription in mouse pancreatic acinar carcinoma 266-6 cells, whereas AP-1 inhibitor reverses this procedure and ameliorates ferroptosis in acute pancreatitis. In addition, the inhibition of FOSL1 has been shown to attenuate VSMC calcification and reactive oxygen species (ROS) generation by enhancing the expression of SLC7A11 and thus inhibits ferroptosis [36]. In the present study, the elevated expression of SLC7A11, FSP1, and GPX4 was observed in the aorta of BAPN-induced AD model mice treated with PCI-34051. Additionally, our findings indicate there is an interaction between HDAC8 and c-JUN, which suggests that PCI-34051 may inhibit VSMC ferroptosis through the AP-1-regulated downstream pathways. However, in subsequent chromatin immunoprecipitation (ChIP)-PCR assays (Fig. S1), we found that PCI-34051 treatment had no effect on the binding of c-JUN to the *GPX4* gene, while it increased the binding to the *SLC7A11* gene regions. Collectively, AP-1 was found to be potentially engaged in the transcriptional regulation of SLC7A11 but not GPX4 in HASMCs. Furthermore, the latest studies have indicated that AP-1 played an important role in the development of AD. Luo et al. [37] found that AP-1 complex mediated the transition of contractile SMCs to both fibro-like SMCs and lipo-SMCs to trigger the development and rupture of AD, and blockade of AP-1 significantly alleviated BAPN-induced thoracic AD in mice. Moreover, AP-1 oligodeoxynucleotides were also reported to reduce aortic elastolysis in a murine model of Marfan Syndrome [38]. The findings of our study indicate that AP-1 is able to mitigate AD by modulating ferroptosis of VSMCs, thereby further elucidating the relationship between AP-1 and AD. Furthermore, we newly identified a potential upstream regulatory target of AP-1, namely HDAC8, whose inhibitor according to our research can ameliorate AD by inhibiting VSMC ferroptosis.

**Figure 8.**
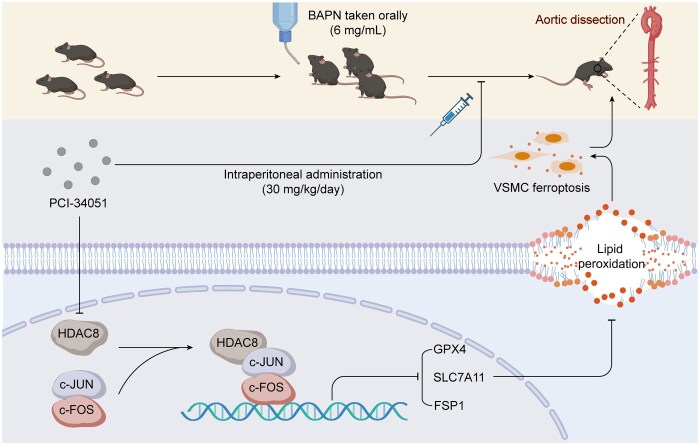
The working model of PCI-34051 regulating HASMC ferroptosis and aortic dissection in mice. PCI-34051 inhibits the ferroptosis of HASMC by affecting the interaction between HDAC8 and AP-1 (c-FOS and c-JUN) and further suppresses the occurrence and development of BAPN-induced aortic dissection in mice. (This figure is created with Biorender).

In the present study, we revealed a novel role of PCI-34051, whereby PCI-34051 suppressed lipid peroxidation and protected VSMCs from ferroptosis. In addition, PCI-34051 could prevent AD and aortic dilation in the BAPN-induced mouse model. Our findings suggest that the HDAC8 inhibitor PCI-34051 may serve as a potential epigenetic therapeutic agent for the prevention and treatment of AD, attenuating pathological progression by suppressing VSMC ferroptosis (Fig. 8). This therapeutic strategy could be further explored for patients with AD associated with elevated HDAC8 activity or ferroptosis activation.

## Research limitations

However, several limitations should be acknowledged:

(i) The efficacy and safety of PCI-34051 need to be validated through primate studies to better model human pathophysiology; (ii) VSMC-specific HDAC8 knockout models are required to definitively establish its mechanistic role in AD pathogenesis. These critical gaps will inform our future investigations aimed at strengthening the translational potential of this research.

## Methods

### Animal experiments

C57BL/6J mice were kept in a specific pathogen-free (SPF) laboratory with constant temperature (25°C) and 12 h-light-dark cycle, adequate normal food and water were supplied with free access. The AD mouse model was established in 3-week-old male and female mice by administering 0.6% BAPN (Sigma-Aldrich, 2079-89-2) in the drinking water for a duration of 4 weeks, as previously described [4]. PCI-34051 (30 mg/kg/d, S2012, Selleck) or DMSO was administrated intraperitoneally every day in the same volume to the BAPN-induced mice. The status of the mice was monitored every day and the dead mice were subjected to necropsy.

### Cell culture and treatments

Human aortic smooth muscle cells (HASMCs) were extracted from the normal aortic tissues which were collected from heart transplant donors as previously described [39]. This study was approved by the Tongji Hospital, Tongji Medical College, Huazhong University of Science and Technology Review Board. The aorta was maintained in cold DME/F12 culture medium (SH30023.01, Hyclone) and the intima and adventitia of aorta were removed under microscope. Subsequently, the media of aorta was cut into 1 mm × 1 mm pieces and distributed evenly in culture flasks. Following a 45-min adherence period, the DME/F12 medium supplied with 10% foetal bovine serum (SH30406.05, Hyclone) and 1% penicillin–streptomycin (15140-122, ThermoFisher Scientific) was added in the flasks. Two weeks later, HASMCs could be observed migrating from the tissue pieces. Once the cells had reached the requisite density, they were digested and transferred to 10 cm culture dishes and passaged every 2–3 days. The 6th–9th generation of HASMCs were used to performed subsequent experiments. Imidazole ketone erastin (IKE, S8877, Selleck) at a concentration of 2.5 µM and cystine deprivation culture medium (CD, DZPYG0257, Boster Biological Technology) were employed to induce ferroptosis in HASMCs in the present study. Additionally, PCI-34051 (S2012, Selleck), a selective inhibitor of HDAC8, was used for cellular treatment at a concentration of 5 µM during the ferroptosis induction. Additionally, to respectively inhibit ferroptosis, apoptosis, autophagic cell death, and necroptosis in HASMCs, the following inhibitors were used: 2.5 μM Ferrostatin-1 (S7243, Selleck), 5 μM Emricasan (S7775, Selleck), 5 mM 3-Methyladenine (HY-19312, MedChemExpress), and 10 μM Necrostatin-1 (S8037, Selleck).

### Plasmids construction and lentivirus infection

For overexpression plasmids, the full-length coding sequences (CDSs) of human *HDAC8*, *c-JUN* and *c-FOS* were amplified through PCR and ligated into the enzymatically cleaved pHAGE lentiviral vector. The primers are presented below: HDAC8 forward primer: 5′-CGACGCGTGCCACCATGGAGGAGCCGGAGGAA-3′, *HDAC8* reverse primer: 5′-CCCTCGAGGACCACATGCTTCAGATTCCCT-3′; *c-JUN* forward primer: 5′-CCGACGCGTGCCACCATGACTGCAAAGATGG-3′, *c-JUN* reverse primer: 5′-CCGCTCGAGAAATGTTTGCAACTGC-3′; *c-FOS* forward primer: 5′-CCGACGCGTGCCACCATGATGTTCTCGGGCTTC-3′, *c-FOS* reverse primer: 5′-CCGCTCGAGCAGGGCCAGCAGCGTGGG-3′. For the *HDAC8-GST* plasmid, the full-length cDNA sequence of *HDAC8* was first amplified by PCR and ligated into the enzymatically cleaved pGEX-4T‑1 vector. The primer sequences used were as follows: *HDAC8‑GST* forward primer: 5′‑TTCCGCGTGGATCCCCGGAATTCATGGAGGAGCCGGAGGAA‑3′; *HDAC8‑GST* reverse primer: 5′‑CAGTCACGATGCGGCCGCTCGAGGACCACATGCTTCAGATTCCCT‑3′. Regarding the knockdown plasmids, short hairpin RNA (shRNA) fragments targeting human HDAC8 were inserted into the pLKO.1 vector. The target sequences are as follows: shRNA-1, 5′-AGTCGCTGGTCCCGGTTTATA-3′; shRNA-2, 5′-TTACGATTGCGACGGAAATTT-3′. The lentivirus was produced as previously described [4]. Briefly, the plasmids together with the package plasmids pMD2. G (12259, Addgene) and psPAX2 (12260, Addgene) were transferred into HEK293T cells through polyethyleneimine (764604, Sigma-Aldrich), then the supernatant culture medium which contained lentivirus was harvested at 24 and 48 h, thereafter, the medium was filtered with a 0.45 µm filter (SLGP033RB, Millipore). HASMCs at an indicated density were infected 24 h with the lentivirus prior to the subsequent experiments.

### Cell viability assay

The Cell Counting Kit-8 (CCK-8, BS350A, Biosharp) was utilized for cell viability assay. HASMCs were transferred into 96-well plates at a density of 8000 cells per well. After adhesion to the wall, the cells were treated with IKE or CD to induce ferroptosis. Subsequently, CCK-8 reagent was mixed with DME/F12 medium in a 1:10 ratio, 100 µL of the work solution was added into each well of the 96-well plates and the plates were incubated at 37°C for 2 h. The optical density (OD) value at 450 nm of each well was measured through a microplate reader (ELx808, BioTek, Winooski, VT).

### Lactate dehydrogenase assay

The lactate dehydrogenase (LDH) produced by HASMCs was quantified through a cytotoxicity LDH assay kit (CK12; Dojindo) to evaluate the extent of cell damage. According to the manufacturer’s instructions, HASMCs were seeded at a density of 8000 cells per well into 96-well plates. Similar to CCK8 assay, after HASMCs were treated with the aforementioned drugs for indicated time, 10 µL lysis buffer was added into the positive control wells and incubated for 30 min at 37°C. Subsequently, the plates were added 100 µL working solution each well and incubated in dark for 25 min. Finally, 50 µL of the stop solution was added to each well. The OD value at 490 nm was measured with the microplate reader.

### Ferrous iron assay

Intracellular Fe^2+^ levels were detected using the FerroOrange fluorescent probe (F374, Dojindo). HASMCs were seeded on coverslips placed in 24-well plates. Following induction of ferroptosis with CD or IKE for a specified duration, the cells were washed three times with HBSS. Subsequently, the HASMCs were incubated in HBSS containing 0.2 μM FerroOrange for 30 min. Cell images were acquired using a fluorescence microscope (Olympus BX53). Quantitative analysis of fluorescence intensity was performed using Image J software.

### Glutathione measurement

Intracellular levels of reduced glutathione (GSH) were measured using a GSH Assay Kit (S0053, Beyotime). HASMCs were seeded in 6-well plates and treated with either PCI-34051 or DMSO, concurrently with ferroptosis induction by CD or IKE for indicated time. Cells were harvested using a protein-removing reagent solution M, followed by two freeze-thaw cycles utilizing liquid nitrogen and a 37°C water bath. After centrifugation at 1000 *g* and 4°C, the supernatant was collected and divided into two aliquots: one for the detection of total intracellular glutathione and the other for oxidized glutathione (GSSG). The aliquot for GSSG measurement required pretreatment with a GSH scavenger reagent. The total glutathione detection working solution was added to both total glutathione and GSSG sample sets. Following a 5-min incubation at room temperature, 0.5 mg/mL NADPH was added to each sample. Absorbance at 412 nm was subsequently measured using a Thermo Scientific Varioskan LUX multimode microplate reader. The GSH content was derived by subtracting the GSSG from the total glutathione.

### Malondialdehyde assay

The malondialdehyde (MDA) level which reflects the degree of intracellular lipid peroxidation was evaluated with an MDA assay kit (S0131M, Beyotime; Abbkine, KTB1050). In brief, HASMCs treated for an indicated time were collected with radioimmunoprecipitation assay (RIPA) lysis buffer. Following ultrasonication and centrifugation (12,000 rpm, 10 min), supernatant of the samples was subjected to protein quantification through a BCA assay kit (23227, ThermoFisher Scientific). Then, 100 µL of supernatant from each sample was mixed with 200 µL of MDA working solution and incubated at 100°C for 15 min. Subsequently, the mixture was centrifuged at 12,000 rpm for 10 min. Finally, 200 µL supernatant was extracted from each mixture and transferred to a 96-well plate and the absorbance at 532 and 600 nm was measured with the microplate reader.

### Reactive oxygen species measurement

Intracellular reactive oxygen species (ROS) levels were quantified using a ROS assay kit (MA0219, Meilunbio). HASMCs were seeded in 24-well plates containing coverslips and incubated with 10 µM DCFH-DA probe for 30 min, followed by ferroptosis induction with either CD or IKE. Fluorescence images of the HASMCs were then acquired using a fluorescence microscope (Olympus BX53), and the ROS fluorescence intensity was quantified with Image J software.

### Flow cytometry

Propidium iodide (PI, P4170, Sigma-Aldrich) staining was performed to evaluate the cell death. HASMCs with different treatments were digested with trypsin and collected in tubes. After centrifugation (1000 rpm, 5 min) and removal of the supernatant, the cells were resuspended in 200 µL binding buffer. Subsequently, each tube was added 100 µL PI staining solution to a final concentration of 5 µg/mL and incubated in dark for 15 min. Finally, the positive staining cells were quantified through a CytoFLEX-3 cytometer (Beckman Coulter). Furthermore, the BODIPY-C11 kit (D3861, ThermoFisher Scientific) was employed for the measurement of cellular lipid peroxidation level. After the designated treatment period, HASMCs were firstly incubated with BODIPY-C11 probes at a concentration of 5 µM for 30 min. Then the cells were digested for collection and centrifuged (1000 rpm, 5 min) in tubes. After resuspension in 300 µL phosphate-buffered saline (PBS), the cell fluorescence intensity was quantified through flow cytometry.

### Immunofluorescence and histology staining

HASMCs were initially inoculated into 24-well plates lined with cell coverslips and treated for indicated time. Following a 15-min fixation with 4% paraformaldehyde, the cells on coverslips were washed with PBS for 3 times and permeabilized with 0.2% Triton X-100 for 20 min. Subsequently, the cells were blocked with 1% bovine serum albumin (BSA, FA016–100 G, Genview) for 1 h and incubated with primary antibody at 4°C overnight. On the following day, the cells on coverslips were incubated with the fluorescent secondary antibody in dark for 2 h and then incubated with 4′, 6-diamidino-2-phenylindole (DAPI, BL105A, Biosharp) for 5 min. The fluorescence images were obtained through a fluorescent microscope (BX53, Olympus).

For histology staining, the aortas of mice were fixed with 4% paraformaldehyde and then embedded in paraffin. The tissues were sectioned at a thickness of 5 μm and subsequently dewaxed in xylene and rehydrated. Haematoxylin and eosin (H&E) and Elastica van Gieson (EVG) staining was performed to observe the morphology and elastic fibre of aortas according to the manufacturer’s protocol. As for immunofluorescence staining, the sections were then immersed in EDTA solution (MVS-0099, MXB biotechnologies) for 20 min at 95°C for antigen retrieval. Then, the sections were blocked in 5% BSA for 1 h and incubated with primary antibody overnight at 4°C. Next day, the sections were incubated with the fluorescent secondary antibody and DAPI. Finally, observation and photography were performed with the fluorescence microscope.

### Real-time PCR

Total intracellular mRNA of HASMCs was extracted using TRIzol Reagent (A33251, Invitrogen). After concentration measurement with a Nanodrop 2000 spectrophotometer, 5 μg of total mRNA was reverse-transcribed into cDNA using a reverse transcription kit (11141ES60, Yeasen). The expression levels of target genes were then analysed by PCR with SYBR Green PCR Master Mix (11201ES08, Yeasen). The primer sequences used were as follows: 18S forward primer: 5′-CTCAACACGGGAAACCTCAC-3′, 18S reverse primer: 5′-CGCTCCACCAACTAAGAACG-3′, HDAC8 forward primer: 5′-CAGAAGGTCAGCCAAGAGGG-3′, HDAC8 reverse primer: 5′-AGTGGCTGGGCAGTCATAAC-3′.

### Western blot

Total proteins from pre-treated HASMCs were extracted with lysis buffer as previously described [40]. The protein samples were denatured at 95°C, then subjected to SDS-polyacrylamide gel electrophoresis and transferred to polyvinylidene fluoride membrane (PVDF, IPVH00010, Millipore) by wet transfer. After being blocked in 5% skim milk for 1 h, the membranes were washed three times with Tris Tween-buffered saline (TBST) and subsequently incubated with the appropriate concentration of specific primary antibody at 4°C overnight. On the subsequent day, after washing for three times with TBST, the membranes were incubated with secondary antibodies at a concentration of 1:10,000 for 2 h at room temperature according to the species of the primary antibody. Finally, the chemiluminescent reagent was applied to the membranes and the protein bands were acquired using the ChemiDoc™ XRS + system (Bio-Rad). Protein concentrations of the various treatment groups were quantified using Image-lab software. The antibodies applied in this study were: β-Actin (AC026, ABclonal), FSP1 (20886-1-AP, Proteintech), GPX4 (ab125066, Abcam), c-JUN (T55290F, ABmart), c-FOS (T56596F, ABmart), Flag (F1804, Sigma-Aldrich), HDAC8 (17548-1-AP, Proteintech), SLC7A11 (26864-1-AP, Proteintech), 4-HNE (MAB3249-SP, Bio-techne), CD86 (13395-1-AP, Proteintech), α-SMA (ab7817, Abcam), α-SMA (GTX100034, Genetex), GST (AE001, Abclonal).

### Co-immunoprecipitation

Co-Immunoprecipitation (Co-IP) was conducted to evaluate the protein interaction between HDAC8 and c-JUN. Following the application of various treatments, HASMCs were lysed with IP lysis buffer and collected in centrifugal tubes. The samples were then sonicated and freeze-thawed three times in liquid nitrogen and room temperature water. Thereafter, the samples were centrifuged (12,000 rpm, 10 min) and the total protein content of the supernatant was quantified through BCA assay kit. Then, 100 µL supernatant from each sample was reserved as input group, while the remainder was incubated with indicated primary antibody and magnetic beads at 4°C overnight. On the next day, the beads were attached to a magnetic holder and washed for five times with washing buffer. Then, 1× SDS loading buffer was added to the beads and each sample was denatured at 95°C for 30 min. Finally, the IP and input samples were subjected to Western blot to examine the immunoprecipitation proteins.

### GST pull-down

The HDAC8-GST fusion plasmid and the GST empty vector were transformed into BL21 competent *E. coli* (C504-02, Vazyme). Single colonies were selected and cultured in LB medium at 37°C with shaking for 2 h, followed by induction with 0.5 mM IPTG (ST098, Beyotime) and continued shaking at 16°C overnight. The bacterial pellet was resuspended in lysis buffer and sonicated to disrupt the cells. The lysate was then centrifuged at 12,000 rpm for 10 min at 4°C, and the supernatant was collected. Protein A/G magnetic beads (B23202, Selleckchem) were added to the supernatant and incubated overnight at 4°C, after which the beads were washed with lysis buffer. Then, total protein of HASMCs was extracted with RIPA lysis buffer and divided into two aliquots, which were incubated overnight at 4°C with GST-HDAC8-bound magnetic beads or GST-only magnetic beads, respectively. The beads were then resuspended in lysis buffer and loading buffer, denatured at 95°C, and subjected to Western-blot analysis.

### Chromatin immunoprecipitation

HASMCs were treated with either CD or CD + PCI-34051 for the indicated duration. Formaldehyde was then added to the culture medium at a final concentration of 1% for cross-linking at room temperature for 10 min. The reaction was quenched by adding 2.5 mol/L glycine solution. After two washes with PBS, the cells were centrifuged at 1000 rpm for 5 min. The supernatant was discarded, and the cell pellet was lysed with cell lysis buffer, followed by sonication. Sonication was performed at 40% power for 30 cycles (3 s ON, 7 s OFF per cycle). The fragmented chromatin was analysed by agarose gel electrophoresis to confirm that the DNA fragment size was within the target range of 400–800 base pairs (bp).

For immunoprecipitation, the samples were incubated overnight at 4°C with antibodies against c-JUN or control immunoglobulin G (IgG), together with Protein A/G magnetic beads. The beads were subsequently washed twice each with Wash Buffer I, Wash Buffer II, and TE buffer. The bound complexes were eluted by resuspending the beads in an elution buffer containing Proteinase K and sodium chloride, followed by overnight incubation at 65°C. The eluted DNA was purified using a DNA purification kit (D0033, Beyotime) and analysed by RT-PCR. The primer sequences used are listed in Table S1.

### RNA sequence

HASMCs were respectively treated with CD + DMSO and CD + PCI-34051 (5 µM) for 10 h. Afterwards, the total RNA of the two groups were collected with TRIzol™ reagent solution (15596026, Invitrogen) and extracted by Novogene Co., Ltd. (Beijing, China) for transcriptome sequencing. The analysis of the differentially expressed genes (DEGs) was performed with a threshold of |log_2_(fold change)| ≥ 0.585 and adjusted *P-*value ≤ 0.05. Based on the DEGs, Gene Ontology (GO) analysis was conducted using the clusterProfiler (v3.16.1) R package.

### Research ethics

All animal experiments in this study were conducted in accordance with the Ethical Guidelines for Animal Experimentation and approved by the Animal Care and Use Committees of Tongji Hospital, Tongji Medical College, Huazhong University of Science and Technology (ethics approval number: TJH-202312035).

### Statistical analysis

In this study, all continuous variables are presented as mean ± SD, ordered categorical variables were presented as the median with ranges. The *t*-test was applied for comparisons between two groups, one-way ANOVA or two-way ANOVA and Tukey’s multiple comparisons test was performed in multiple group comparisons. The Mann–Whitney *U* test was utilized for analysis of the ordered categorical variables. All the data were analysed with GraphPad Prism 8 software, *P *< 0.05 is considered to be statistically significant.

## Supplementary Material

lnag013_Supplementary_Data

## Data Availability

All data supporting the findings of this study are included in this published article and its supplementary information files.
