# Supplementary material for: HDAC8-selective inhibitor PCI-34051 protects against aortic dissection by attenuating ferroptosis of vascular smooth muscle cells
Source: Life Med. 2026 Apr 17;5(3):lnag013. doi: 10.1093/lifemedi/lnag013 (PMC13250666; doi:10.1093/lifemedi/lnag013)
Supplement: lnag013_Supplementary_Data [file lnag013_supplementary_data.zip › Revised supplementary data_PE.docx]

**HDAC8-selective inhibitor PCI-34051 protects against aortic dissection by attenuating ferroptosis of vascular smooth muscle cells**

Jiannan Ye^1,*^, Juan Shi^1,*^, Xin Yi^2^, Jingjie Chen^1^, Yi He^1^, Bo Huo^1^, Hanshen Luo^1^, Shibin Chen^1^, Xiang Wei^1,3^, Ding-Sheng Jiang^1,3,#^, Ze-Min Fang^1,#^

^1^Division of Cardiovascular Surgery, Tongji Hospital, Tongji Medical College, Huazhong University of Science and Technology, Wuhan 430030, China

^2^Department of Cardiology, Renmin Hospital of Wuhan University, Wuhan 430030, China

^3^Key Laboratory of Organ Transplantation, Ministry of Education; NHC Key Laboratory of Organ Transplantation; Key Laboratory of Organ Transplantation, Chinese Academy of Medical Sciences, Wuhan 430030, China

^*^These authors contributed equally to this work.

**^#^**Correspondence: jds@hust.edu.cn (D.S.J.), zmfang@hust.edu.cn (Z.M.F.)

**Supplemental Table and Figures**

**Supplemental Table S1. Primers for CHIP-PCR detection.**

| Gene | Sequence 5’→3’ |
| --- | --- |
| GPX4 | F: ATTAGAGTCCAGGCGAGGGC  R: TTCCTCCTGCAACTTCACC |
| SLC7A11-1 | F: GCTCTGTCCATGTTCCCACA  R: TGCTCATCAGTGACAGATTGGA |
| SLC7A11-2 | F: GGGTGGATCACGAGGTCAAG  R: CAGGCTGGAGTGCAGTGG |

**Figure S1. Chromatin Immunoprecipitation (ChIP) analysis of the effects of PCI-34051 on c-JUN binding to the gene region of GPX4 and SLC7A11.**

ChIP assays were performed in HASMCs treated with CD or CD + PCI-34051. Immunoprecipitation was carried out using an anti-c-JUN antibody or normal rabbit IgG (negative control). The enrichment of c-JUN at the regions of the GPX4 and SLC7A11 genes was quantified by qPCR. Data are presented as percentage of input (*n* = 3 per group). Values are means ± SD; ***p* < 0.01, **p* < 0.05.

**Figure S2. Negative IgG control for immunofluorescence staining.**

Representative image of aortic tissues stained with normal IgG (same species and isotype as the primary antibody) followed by the corresponding fluorescent secondary antibody.
