# Supplementary figures and images for: HDAC8-selective inhibitor PCI-34051 protects against aortic dissection by attenuating ferroptosis of vascular smooth muscle cells
Source: Life Med. 2026 Apr 17;5(3):lnag013. doi: 10.1093/lifemedi/lnag013 (PMC13250666; doi:10.1093/lifemedi/lnag013)

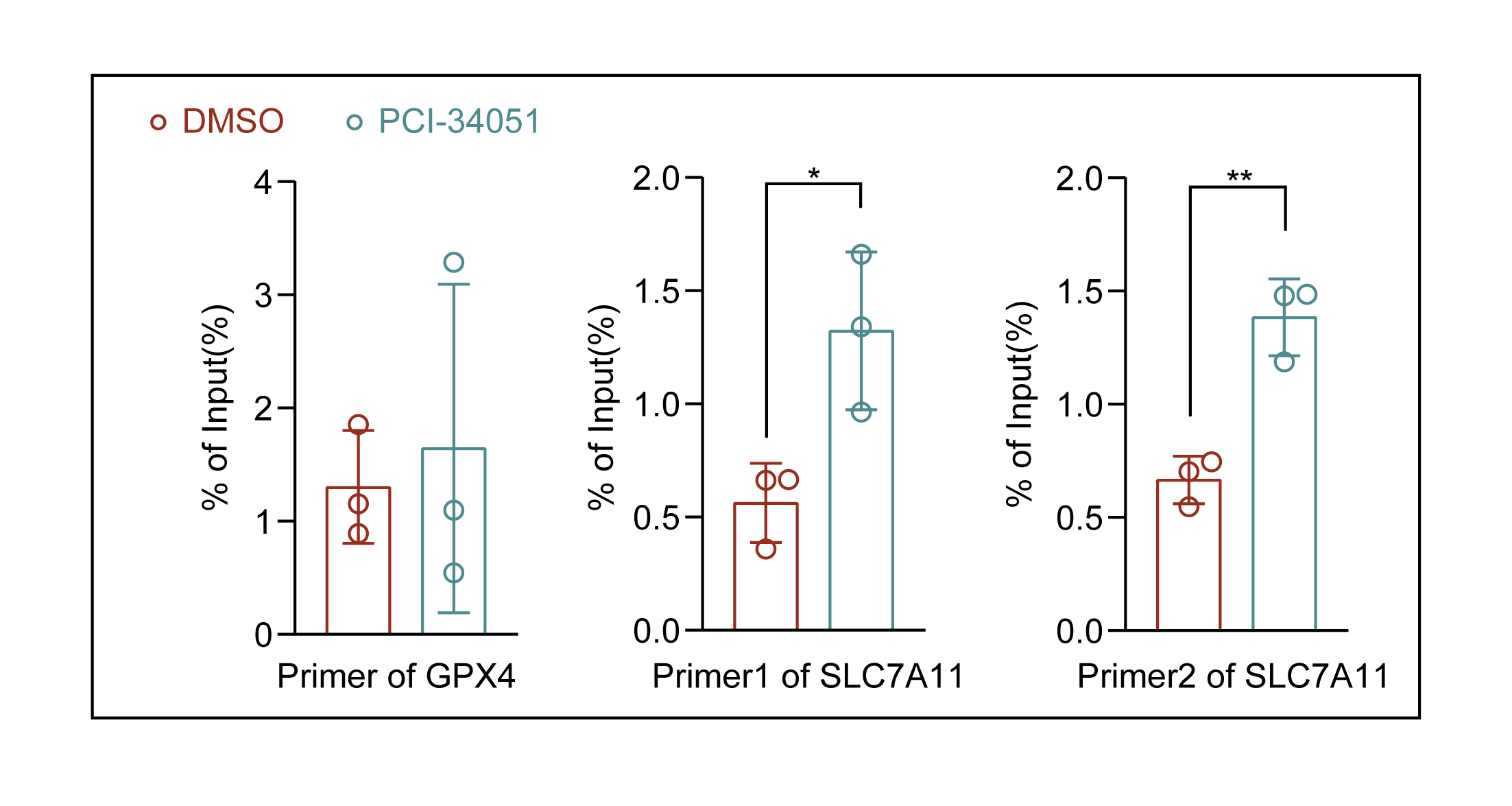

Supplement: lnag013_Supplementary_Data [file lnag013_supplementary_data.zip › Figure S1.tif]

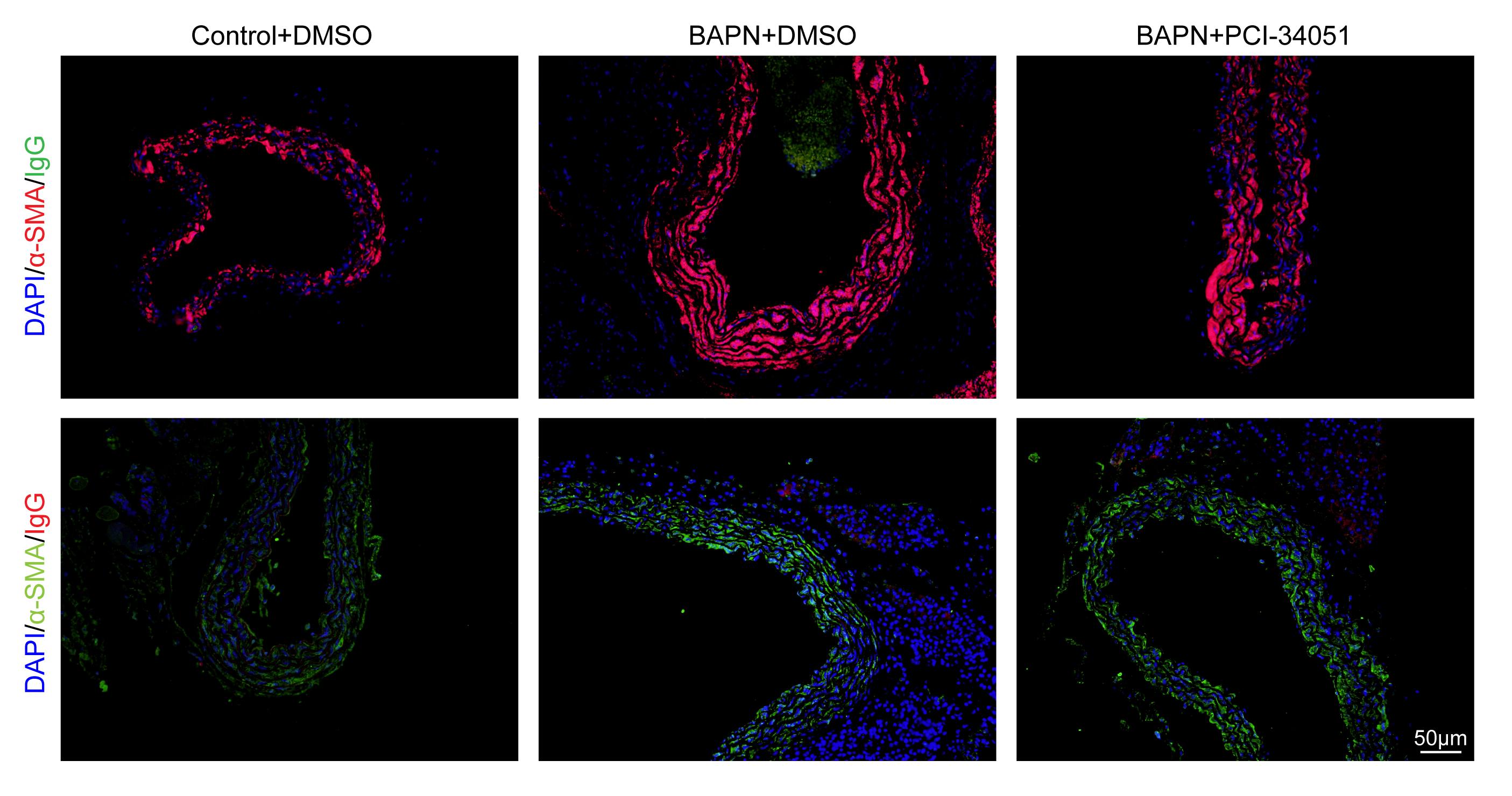

Supplement: lnag013_Supplementary_Data [file lnag013_supplementary_data.zip › Figure S2.tif]
